# The Intracellular DNA Sensor IFI16 Gene Acts as Restriction Factor for Human Cytomegalovirus Replication

**DOI:** 10.1371/journal.ppat.1002498

**Published:** 2012-01-26

**Authors:** Grazia Rosaria Gariano, Valentina Dell'Oste, Matteo Bronzini, Deborah Gatti, Anna Luganini, Marco De Andrea, Giorgio Gribaudo, Marisa Gariglio, Santo Landolfo

**Affiliations:** 1 Department of Public Health and Microbiology, University of Turin, Turin, Italy; 2 Department of Clinical and Experimental Medicine, University of Piemonte Orientale, Novara, Italy; University of North Carolina at Chapel Hill, United States of America

## Abstract

Human interferon (IFN)-inducible IFI16 protein, an innate immune sensor of intracellular DNA, modulates various cell functions, however, its role in regulating virus growth remains unresolved. Here, we adopt two approaches to investigate whether IFI16 exerts pro- and/or anti-viral actions. First, the IFI16 gene was silenced using specific small interfering RNAs (siRNA) in human embryo lung fibroblasts (HELF) and replication of DNA and RNA viruses evaluated. IFI16-knockdown resulted in enhanced replication of Herpesviruses, in particular, Human Cytomegalovirus (HCMV). Consistent with this, HELF transduction with a dominant negative form of IFI16 lacking the PYRIN domain (PYD) enhanced the replication of HCMV. Second, HCMV replication was compared between HELFs overexpressing either the IFI16 gene or the LacZ gene. IFI16 overexpression decreased both virus yield and viral DNA copy number. Early and late, but not immediate-early, mRNAs and proteins were strongly down-regulated, thus IFI16 may exert its antiviral effect by impairing viral DNA synthesis. Constructs with the luciferase reporter gene driven by deleted or site-specific mutated forms of the HCMV DNA polymerase (UL54) promoter demonstrated that the inverted repeat element 1 (IR-1), located between −54 and −43 relative to the transcription start site, is the target of IFI16 suppression. Indeed, electrophoretic mobility shift assays and chromatin immunoprecipitation demonstrated that suppression of the UL54 promoter is mediated by IFI16-induced blocking of Sp1-like factors. Consistent with these results, deletion of the putative Sp1 responsive element from the HCMV UL44 promoter also relieved IFI16 suppression. Together, these data implicate IFI16 as a novel restriction factor against HCMV replication and provide new insight into the physiological functions of the IFN-inducible gene IFI16 as a viral restriction factor.

## Author Summary

Only recently, intrinsic cellular-based defense mechanisms which give cells the capacity to resist pathogens have been discovered as an essential component of immunity. However, unlike the innate and adaptive branches of the immune system, intrinsic immune defenses are mediated by cellular restriction factors that are constitutively expressed and active even before a pathogen enters the cell. The protein family HIN-200 may act as sensors of foreign DNA and modulate various functions such as growth, apoptosis, and senescence. Here we show that, in the absence of functional IFI16, the replication of some Herpesviruses and in particular of Human Cytomegalovirus (HCMV) is significantly enhanced. Accordingly, IFI16 overexpression strongly inhibited HCMV replication. Accumulation of viral DNA copies was down-regulated along with expression of early and late viral gene expression suggesting that IFI16 inhibits viral DNA synthesis. Using transient transfection, luciferase, gel shift assay, and chromatin immunoprecipitation, we demonstrate that IFI16 suppresses the transcriptional activity of the viral DNA polymerase gene (UL54) and the UL44 gene, also required for viral DNA synthesis. The finding that the nuclear DNA sensor IFI16 controls virus growth represents an important step forward in understanding the intrinsic mechanisms that drive viral infections sustained by DNA viruses such as Herpesviruses.

## Introduction

Human Cytomegalovirus (HCMV) is a β-Herpesvirus that commonly and persistently infects humans [1]–[2]. HCMV does not constitute a serious threat to immunocompetent individuals, but causes life-threatening complications in individuals with suppressed immune systems, such as patients with AIDS, cancer patients undergoing chemotherapy, and organ transplant recipients treated with immunosuppressants [3]. Viral gene expression in HCMV undergoes sequential regulation, which leads to the occurrence of induction and repression cycles in the immediate early (IE), early (E), and late (L) phases of viral replication. IE1 and IE2 induce the expression of early protein, mediate G1/S cell cycle arrest and host replication shut-off [4]–[5].

Many mammals, including humans, are equipped with genes encoding so-called “restriction factors” that provide considerable resistance to viral infection [6]. Such intrinsic immune mechanisms are highly important as they provide an antiviral frontline defense mediated by constitutively expressed proteins, already present and active before a virus enters a cell [7]–[8]. These intrinsic immune mechanisms were initially discovered as being active against retroviruses and involve the APOBEC3 class of cytidine deaminases, a large family of proteins termed the TRIM family, and tetherin, an interferon-inducible protein whose expression blocks the release of HIV-1. It has recently emerged, however, that such intrinsic immune mechanisms are also active against other viruses, such as Vesicular Stomatitis Virus, Filoviruses, Influenza Virus, and Hepatitis C Virus [9]. Moreover, four cellular proteins – promyelocitic leukemia protein (PML), hDaxx, Sp100 [10], and viperin – have been identified as restriction factors involved in mediating intrinsic immunity against HCMV infection [11]. PML and hDaxx are components of a subnuclear structure known as nuclear domain 10 (ND10) or PML nuclear bodies. Direct evidence for their antiviral role has been obtained from infection studies using cells devoid of intact ND10. Primary human fibroblasts depleted of PML using small interfering RNA (siRNA) significantly increased the plaque forming efficiency of HCMV as a result of an augmented immediate-early (IE) gene expression [12]. hDaxx represses HCMV IE gene expression [11], [13], [14] and replication [12] through the action of a histone deacetylase (HDAC), thereby inducing a transcriptionally inactive chromatin state around the major IE enhancer/promoter (MIEP) of HCMV [13]–[14]. Together, these findings revealed that ND10 proteins, PML and hDaxx, act as cellular restriction factors that are able to induce silencing of HCMV gene expression, thus controlling virus replication.

Of the various interferon-inducible proteins, the p200 family of proteins, now designated the PYHIN family, consists of a group of homologous human and mouse proteins that have an N-terminal PYRIN domain (PYD) and one or two partially conserved 200 amino acid long C-terminal domains (HIN domain). These proteins display multifaceted activity due to their ability to bind to various target proteins (e.g. transcription factors, signaling proteins, and tumor suppressor proteins) and modulate different cell functions. Increasing evidence supports a role for these proteins as regulators of various cell functions, including proliferation, differentiation, apoptosis, senescence, and inflammasome assembly, as well as in the control of organ transplants (reviewed in [15]–[16]). More recently, two members of the PYHIN family, namely AIM2 and IFI16, have been shown to bind to and function as pattern recognition receptors (PRR) of virus-derived intracellular DNA [17]–[20]. In particular, IFI16 has been shown to interact with the adaptor molecule ASC and procaspase-1 to form a functional inflammasome during Kaposi Sarcoma-Associated Herpesvirus (KSHV) infection. KSHV gene expression is required for inflammasome activation and IFI16 colocalizes with the KSHV genome in infected cell nuclei [20]. Moreover, the induction of IRF3 and NF-κB-dependent genes by HSV-1 infection of RAW264.7 cells is strongly impaired by siRNA specific for p204, the mouse ortholog of IFI16 [19]. However, although many different functions have been ascribed to these proteins, their roles as antiviral restriction factors have yet to be investigated, whilst such roles have long been established for other IFN-inducible proteins, such as, PKR, 2′-5′ oligoadenylate synthetase, and MxA [21]–[22].

In the present study, by either silencing or overexpressing the IFI16 protein, we demonstrate for the first time that IFI16 acts as a restriction factor for Human Cytomegalovirus (HCMV) replication. Transfection and electrophoretic mobility shift assay (EMSA) experiments, performed using nuclear extracts from HCMV infected cells, showed that the promoter of the DNA polymerase gene (UL54) is the target of IFI16-induced viral suppression. Finally, biochemical and immunochemical analyses reveal that IFI16 inhibits DNA polymerase gene activity by preventing the binding of transcription factor Sp1 to the −54/−43 IR-1 promoter element.

## Results

### IFI16 protein specifically inhibits replication of Herpesviruses but not of other viruses

**Figure 1:**
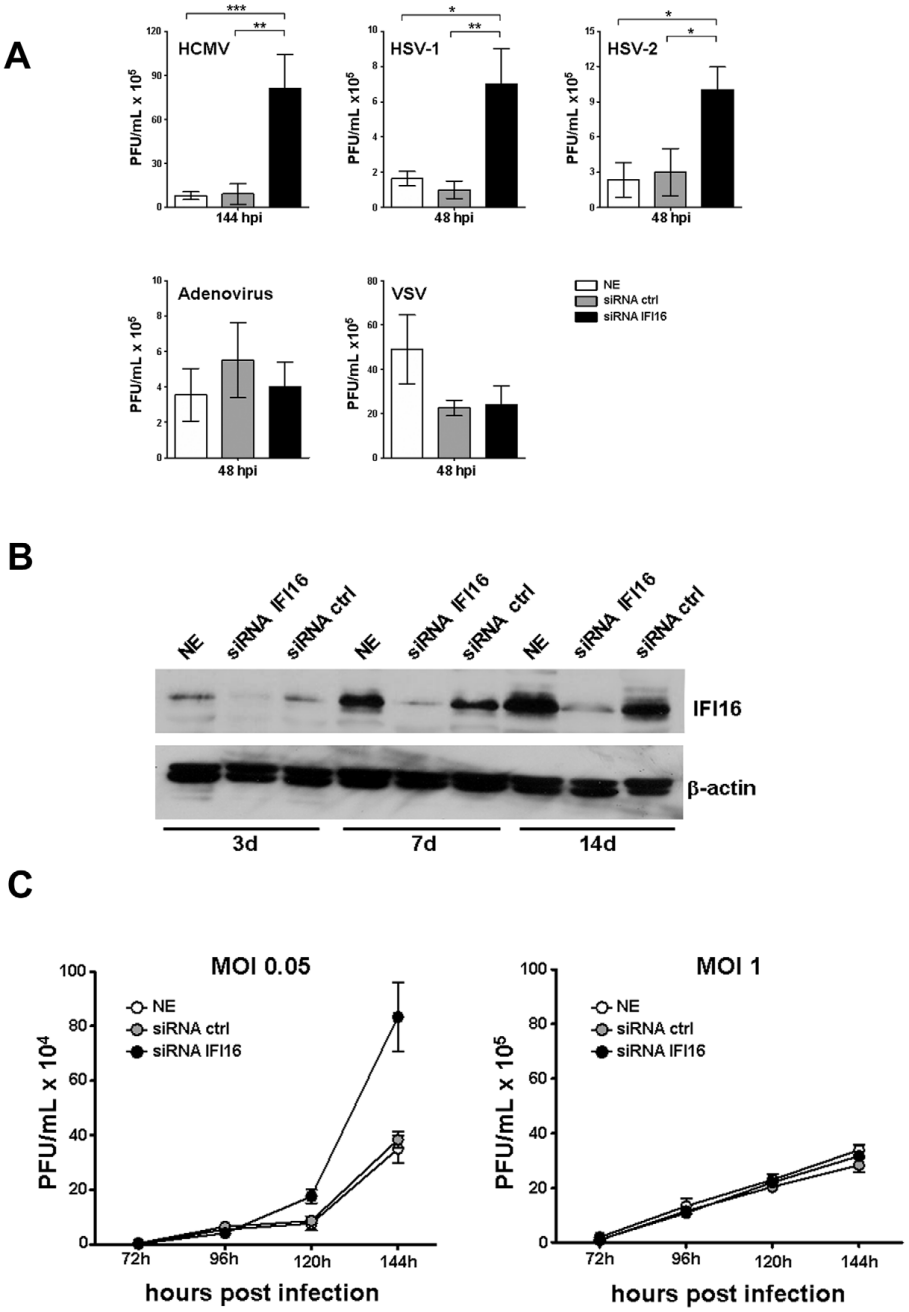
IFI16 protein is a negative regulator of Herpesvirus replication. A) HELFs were electroporated with a mixture of four different small interfering RNAs (siRNA IFI16), scrambled control siRNA (siRNA ctrl), or left not electroporated (NE), and then infected with the indicated viruses at a multiplicity of 0.05 PFU/cell. Cell-free supernatants were harvested on the indicated hours post infection (hpi) and virus amounts determined by plaque assays. The data shown are the average of three experiments ± SD (*p<0.05, **p<0.01, ***p<0.001, one-way ANOVA followed by Bonferroni's post test). B) HELFs were transfected with siRNA IFI16 or siRNA ctrl or left not electroporated (NE) and IFI16 expression assayed by Western blotting on the indicated days (d) with anti-IFI16 polyclonal Abs. β-actin was included as a loading control. C) HELFs were treated with siRNA as described for panel A and then infected with HCMV at an MOI of 0.05 (left panel) or 1 (right panel) PFU/cell. Cell-free supernatants were harvested on the indicated hours post infection and the virus amounts determined by plaque assays. The data shown are the average of three experiments ± SD.

To determine the effects of IFI16 silencing on virus replication, IFI16-knockdown HELFs were infected with HCMV (AD169 strain), HSV-1, HSV-2, ADV, or VSV at an MOI of 0.05. As shown in Figure 1A, the replication of HSV at 48 hpi and of HCMV at 144 hpi was increased in IFI16-silenced HELFs. In contrast, the replication of a clinical isolate of Adenovirus and that of a VSV laboratory strain was not significantly affected (Figure 1A). Consistent with previous data, Western blot analysis confirmed that the electroporation of HELFs with four IFI16 specific siRNAs knocked IFI16 expression down by more than 90% for at least 14 days (Figure 1B).

**Figure 2:**
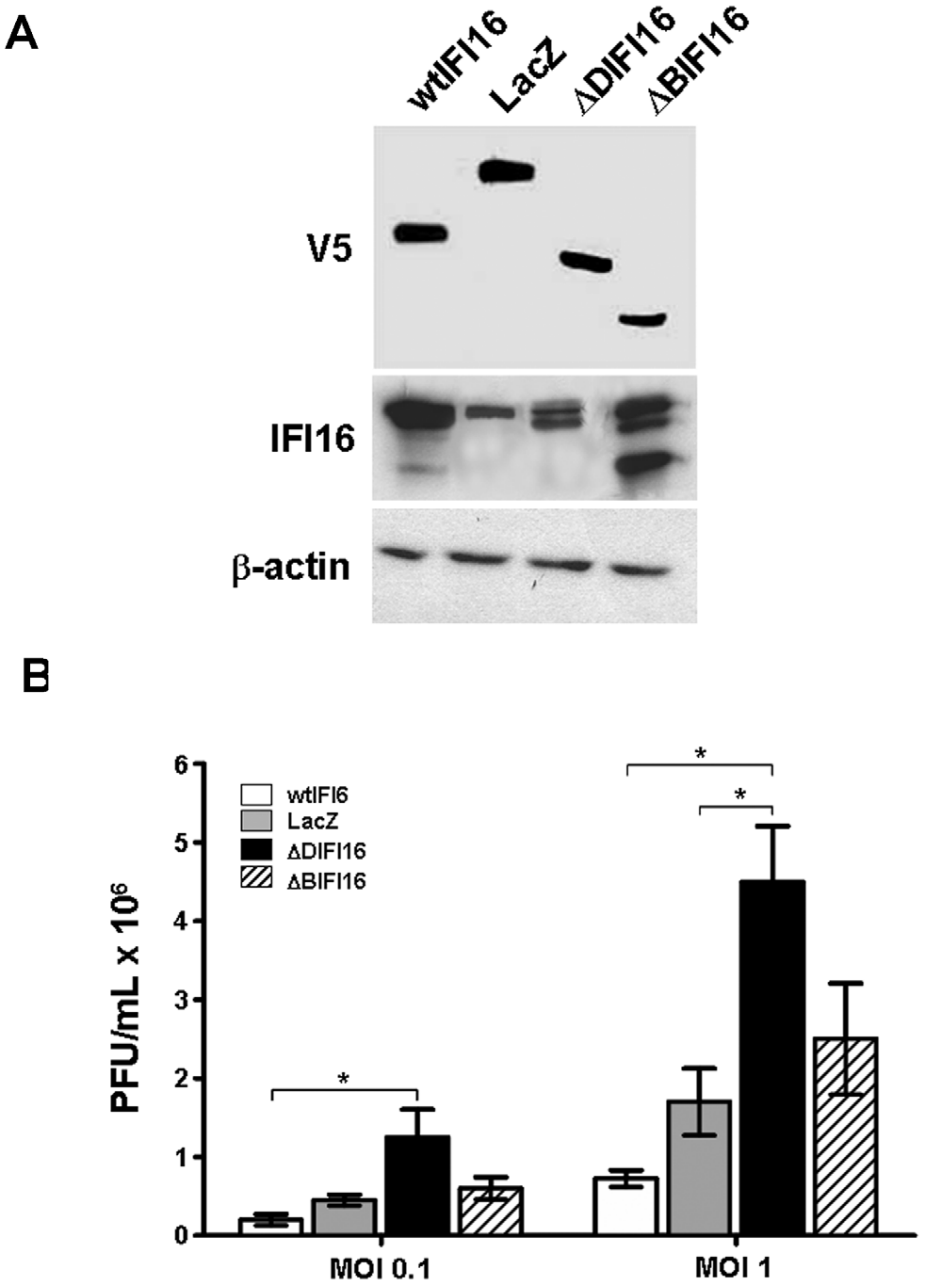
Effect of dominant negative IFI16 (dnIFI16) overexpression on Herpesvirus growth. A) Western blot analysis was carried out to detect IFI16 and V5 expression in HELFs stably transduced with the recombinant Lentivirus carrying the full-length IFI16 (wtIFI16), mutated forms of IFI16 (dnIFI16), lacking the PYD domain (ΔPYDIFI16, indicated as ΔDIFI16), the HIN-B domain (ΔHIN-BIFI16, indicated as ΔBIFI16) or expressing the LacZ transgene as negative control (LacZ). β-actin immunodetection was used to control for equal loading. B) HELFs carrying wtIFI16, ΔDIFI16, ΔBIFI16 or the control LacZ gene were infected with HCMV at the indicated MOI. Viral supernatants were collected at 96 hours post infection (hpi) and analyzed by standard plaque assay. The data shown are the average of three experiments ± SD (*p<0.05, one-way ANOVA followed by Bonferroni's post test).

To provide further evidence supporting the physiological relevance of IFI16 in the control of Herpesvirus replication, we generated HELFs overexpressing wild type (wt) or mutated IFI16 proteins using recombinant lentiviral vectors, or the LacZ gene as a control. These V5-tagged IFI16 proteins bear deletions between residues 1–83 (ΔPYDIFI16, indicated as ΔDIFI16) or between residues 515–710 (ΔHIN-BIFI16, indicated as ΔBIFI16), respectively, and thus held the potential to inhibit the activity of the endogenous counterpart. Expression of the exogenous V5-tagged IFI16 was confirmed using anti-V5 antibodies that recognized proteins of about 82 kDa (wtIFI16), 70 kDa (ΔDIFI16) or 60 kDa (ΔBIFI16), and 121 kDa in the control (LacZ) (Figure 2A).When overexpressed in stably-transfected cell lines, both ΔDIFI16 and ΔBIFI16 decreased the ability of full length IFI16 (AdV IFI16) to induce proinflammatory molecules and to trigger caspase-3 and 7 activity (Figure S1 panel A and B respectively). These results suggest that the mutant IFI16 proteins may behave as dominant negative (dn) towards the endogenous counterpart.

The effects of ΔDIFI16 and ΔBIFI16 deleted mutants on viral growth were then examined by comparing the ability of HELFs transfected with either the mutated forms of IFI16 or the full length protein to support HCMV complete replication. In line with the results of the previous experiments that used siRNAs to silence IFI16, expression of the ΔDIFI16 protein substantially increased the extent of HCMV replication (Figure 2B) at both the MOIs used. Expression of ΔBIFI16 showed a level of virus yield similar to that observed in HELFs transduced with the LacZ gene, whereas overexpression of full length IFI16 reduced virus replication. Altogether, these results demonstrate that reducing IFI16 activity with ΔDIFI16 consistently increases the rate of HCMV replication, whereas overexpression of the full length protein down-regulates its replication, implying IFI16 as a novel restriction factor in the replicative cycle of Herpesviruses.

However, these above results are in apparent contrast with those of Cristea et al. [23] and those previously published by Rolle et al. [24] with the IFI16 mouse homolog Ifi204, who found that knocking down IFI16 or Ifi204 expression caused a delay in the accumulation of infectious CMV progeny, but no net reduction in its replication. To explain these discrepancies, we performed kinetic experiments in which IFI16-knockdown HELFs (i.e. IFI16-silenced using siRNA) were infected with HCMV at two different MOI (0.05 and 1, respectively) and the viral yield evaluated at different time points post infection (hpi). At 96 hpi, the number of infectious particles measured in the IFI16-knockdown cells (siRNA IFI16) infected at an MOI of 0.05 was actually decreased compared to that of HELFs electroporated with control siRNA (siRNA ctrl) or left not electroporated (NE) (Figure 1C, left panel). At 120 hpi, the number of plaque forming units (PFU) in the absence of IFI16 expression started to be higher than that observed in the control HELF cultures electroporated with siRNA ctrl or sham-electroporated. In contrast, at 144 hpi, the accumulation of HCMV progeny exhibited an ∼3- fold increased yield in the absence of IFI16 expression. At the higher MOI, no differences in virus yield were observed irrespective of the level of IFI16 expression (Figure 1C, right panel). Thus, in line with the results of Cristea et al. [23] and Rolle et al. [24], our findings indicate that Herpesvirus replication, and in particular that of HCMV, may be impaired by IFI16 silencing in the first hours after infection at the lower MOI, but as virus replication progresses this impairment becomes lost as shown by increased viral yields compared to controls at the later time points p.i. At higher MOI, however, the relevance of IFI16 in the control of HCMV replication appears to be less.

HCMV is able to replicate *in vivo* and *in vitro* in many different host cells including vascular endothelial cells, epithelial cells, connective tissue cells, hepatocytes, and various leukocyte populations (reviewed in [25]). Since fibroblasts are not the most important target cells *in vivo*, we evaluated the effect of IFI16 silencing on HCMV replication in human umbilical vein endothelial cells (HUVEC), a cell system considered to be more pertinent for HCMV replication *in vivo*. For this purpose, HUVEC were electroporated with siRNA IFI16 or siRNA ctrl or left not electroporated (NE) and then infected with the endotheliotropic HCMV strain VR1814 at MOI of 0.1 or 1. As shown in Figure S2, and in line with the results obtained using fibroblasts, replication of the VR1814 strain was significantly increased at an MOI of 1 compared to that observed in cells treated with siRNA ctrl (∼7 fold increase). In contrast to our previously observations in fibroblasts, at the lower MOI the level of virus replication in endothelial cells in the absence of IFI16 was still different but at lower degree from that observed in controls. This difference may be explained by the fact that the efficiency of infection and replication of the VR1814 strain in endothelial cells is much lower than that of the AD169 virus strain in fibroblasts [26]. Taken together, these results demonstrate that IFI16 may also restrict HCMV replication in physiologically relevant target cells *in vivo* such as endothelial cells.

### IFI16 overexpression impairs HCMV replication by inhibiting early and late viral gene expression

**Figure 3:**
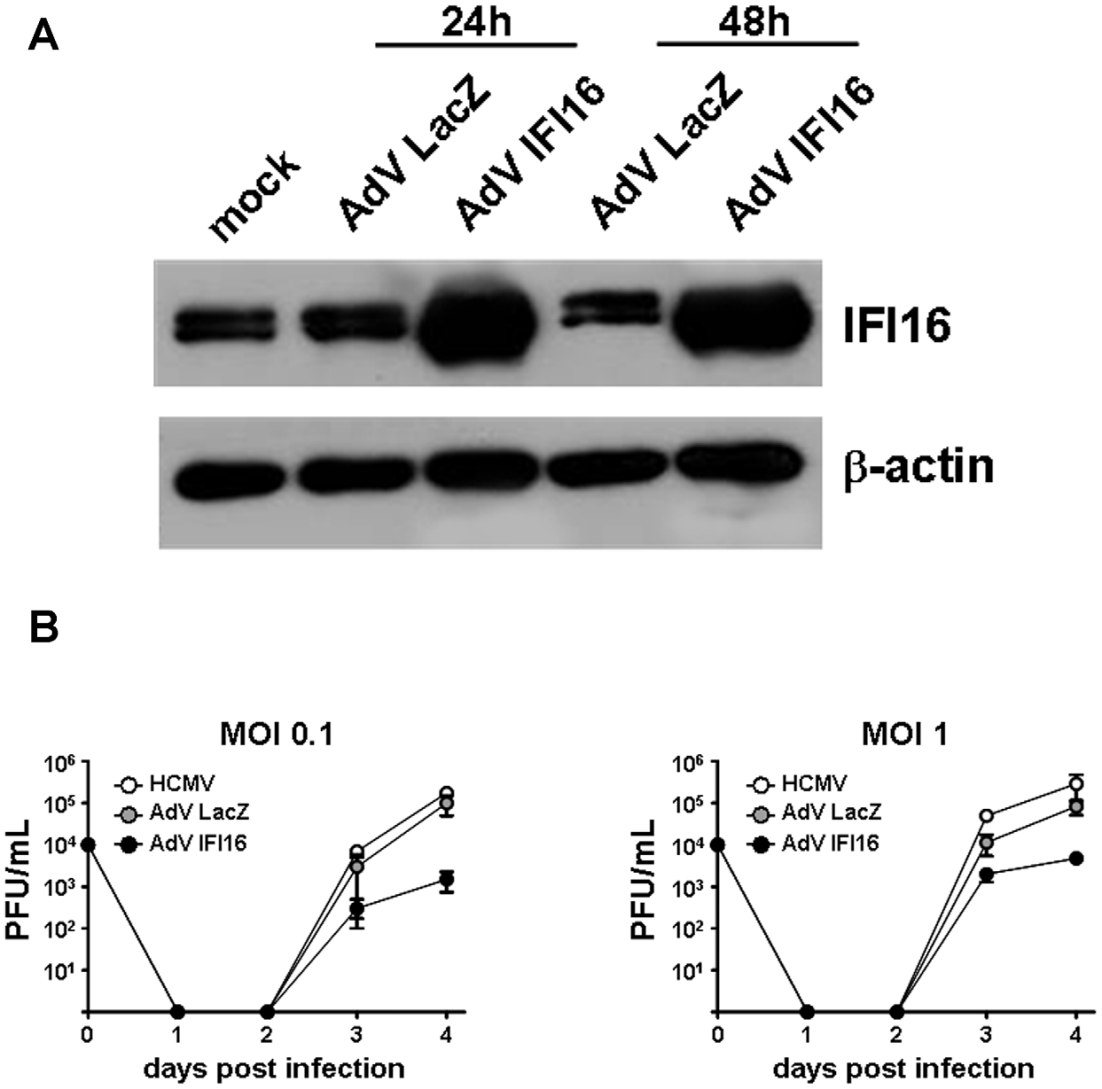
Overexpression of IFI16 reduces HCMV replication by inhibiting viral early and late gene expression. A) Kinetics of Adenovirus-mediated IFI16 overexpression. HELFs were infected with AdV IFI16, AdV LacZ (MOI of 200 PFU/cell), or mock-infected. At the indicated hours post infection (h), total cell extracts were prepared and subjected to Western blot analysis using anti-IFI16 polyclonal Ab. β-actin served as the internal control. B) HELFs were infected with AdV IFI16, AdV LacZ (MOI of 200 PFU/cell), or left untreated. After 24 hours, cells were infected with HCMV at an MOI of 0.1 (left panel) or 1 (right panel) PFU/cell. Cell-free supernatants were harvested on the indicated days post infection and the amounts of HCMV were determined by standard plaque assay. The data shown are the average of three experiments ± SD.

To gain more insight into the antiviral activity of IFI16, we focused on the HCMV model. HELFs were infected with AdV IFI16 or AdV LacZ at an MOI of 200 and the IFI16 protein content monitored by Western blot analysis. As shown in Figure 3A, IFI16 protein levels started to increase as early as 24 hpi and continued to increase until 48 hpi. When HELFs overexpressing wt IFI16 for 24 h were infected with HCMV at an MOI of 0.1, a ∼2.5 log decrease in viral production was observed on day 4, compared to HELFs left untransduced or transduced with the control AdV LacZ gene (Figure 3B, left panel). A similar pattern was observed at the higher MOI of 1, but with lower levels of virus growth suppression (∼1 log reduction) (Figure 3B, right panel). Thus, consistent with the previous findings, which showed increased virus replication in the absence of IFI16, the overexpression of IFI16 strongly inhibits HCMV replication.

**Figure 4:**
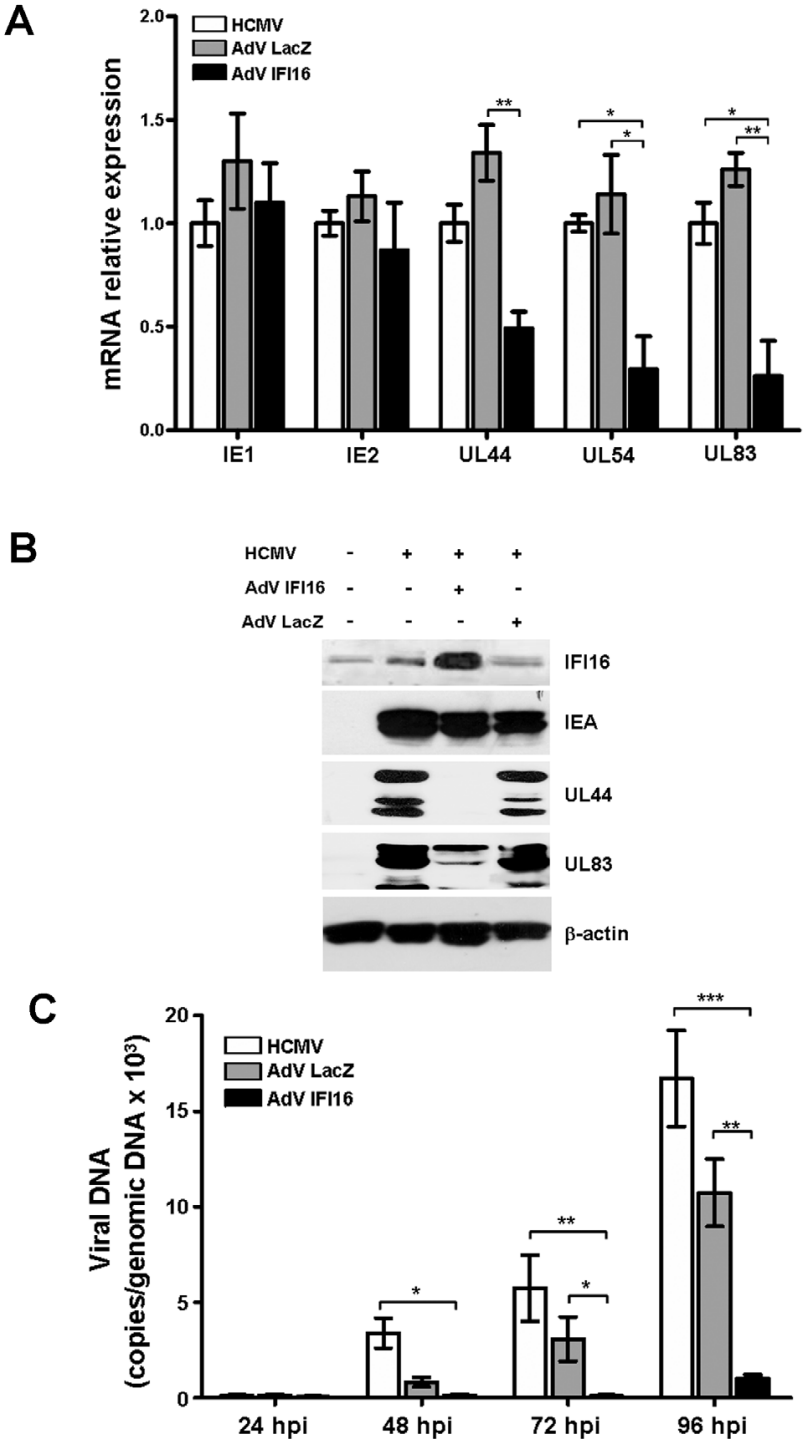
IFI16 impairs early and late, but not immediate early gene expression. A) HELFs were infected with AdV IFI16, AdV LacZ (MOI of 200 PFU/cell), or left untreated. After 24 hours, cells were infected with HCMV at an MOI of 1 PFU/cell. Total RNA was isolated at 24 hours post infection and assayed by quantitative real-time PCR to determine the relative levels of viral transcripts. Levels of viral mRNA are presented normalized to the levels of cellular β-actin. The data shown are the average of three experiments ± SD (*p<0.05, **p<0.01, one-way ANOVA followed by Bonferroni's post test). B) Cells were infected as described in the legend for panel A. Thirty µg of protein were analyzed by Western blotting for viral protein expression at 48 hours post infection and β-actin was used as internal control. C) Cells were infected as described in the legend for panel A. Viral DNA was isolated at the indicated hours post infection (hpi) and analyzed by quantitative real-time PCR, whereby primers amplified a segment of the E1 gene to determine the number of viral DNA genomes per nanogram of cellular reference DNA (18S rRNA gene). The data shown are the average of three experiments ± SD (*p<0.05, **p<0.01, ***p<0.001, one-way ANOVA followed by Bonferroni's post test).

To investigate the molecular basis of the antiviral activity of IFI16, we examined the effects of its overexpression on different phases of the HCMV replication cycle. To this purpose, HELFs were infected with AdV IFI16 or AdV LacZ (MOI of 200) or left uninfected (mock), and infected 24 h later with HCMV (MOI of 1) for a further 24 h. The amounts of IE (IE1 and IE2), UL44, UL54, and UL83 transcripts were then assessed by quantitative real-time PCR as markers of IE (IE1 and IE2), E (UL44 and UL54), and L (UL83) mRNAs (Figure 4A). According to Cristea et al [23] results, we saw no difference in expression of the products of immediate early genes (IE1 and IE2) between IFI16-overexpressing and LacZ- or mock-infected cells. In contrast, mRNA synthesis of early (UL44 and UL54) and early-late (UL83) genes was significantly reduced (2-, 6- and 6- fold respectively) in the cells expressing IFI16. Total protein extracts were then analyzed for their content of immediate early (IE), early (UL44), and early-late (UL83) proteins by immunoblotting with specific antibodies. As shown in Figure 4B, the expression of the HCMV UL44 and UL83 proteins was strongly impaired in AdV IFI16-infected HELFs compared to that seen in control cells, mirroring the results obtained at the mRNA level. The expression of these proteins is indispensable for productive HCMV infection (reviewed in [1]). A possible explanation for these findings is that IFI16 may exert its antiviral effect by inhibiting the synthesis or function of an HCMV-encoded component critical for viral DNA synthesis and/or maturation. Further support to this hypothesis comes from the experiments in which viral DNA synthesis was measured in HELFs transduced with IFI16 or LacZ genes for 24 h and then infected with HCMV. As shown in Figure 4C, starting at 48 hpi and continuing at later time points (72 and 96 hpi), a significant decrease in the number of viral DNA copies was observed in HELFs transduced with the IFI16 gene compared to cells left untransduced or transduced with the LacZ gene.

Taken together, these results indicate that IFI16 reduces HCMV replication by inhibiting the expression of E and L genes required for the viral DNA synthesis and completion of the viral productive cycle.

### IFI16 overexpression inhibits the transcription of the HCMV UL54 and UL44 gene in the context of viral infection

Previous studies have shown that cotransfection of IFI16 and a CAT reporter gene containing the wild type UL54 promoter results in a dose-dependent decrease in reporter activity [27] suggesting an interplay between IFI16 and transcription factors responsible for UL54 promoter activation. These observations were made, however, in uninfected cells using the UL54 essential promoter as a target of cellular transcription factors.

**Figure 5:**
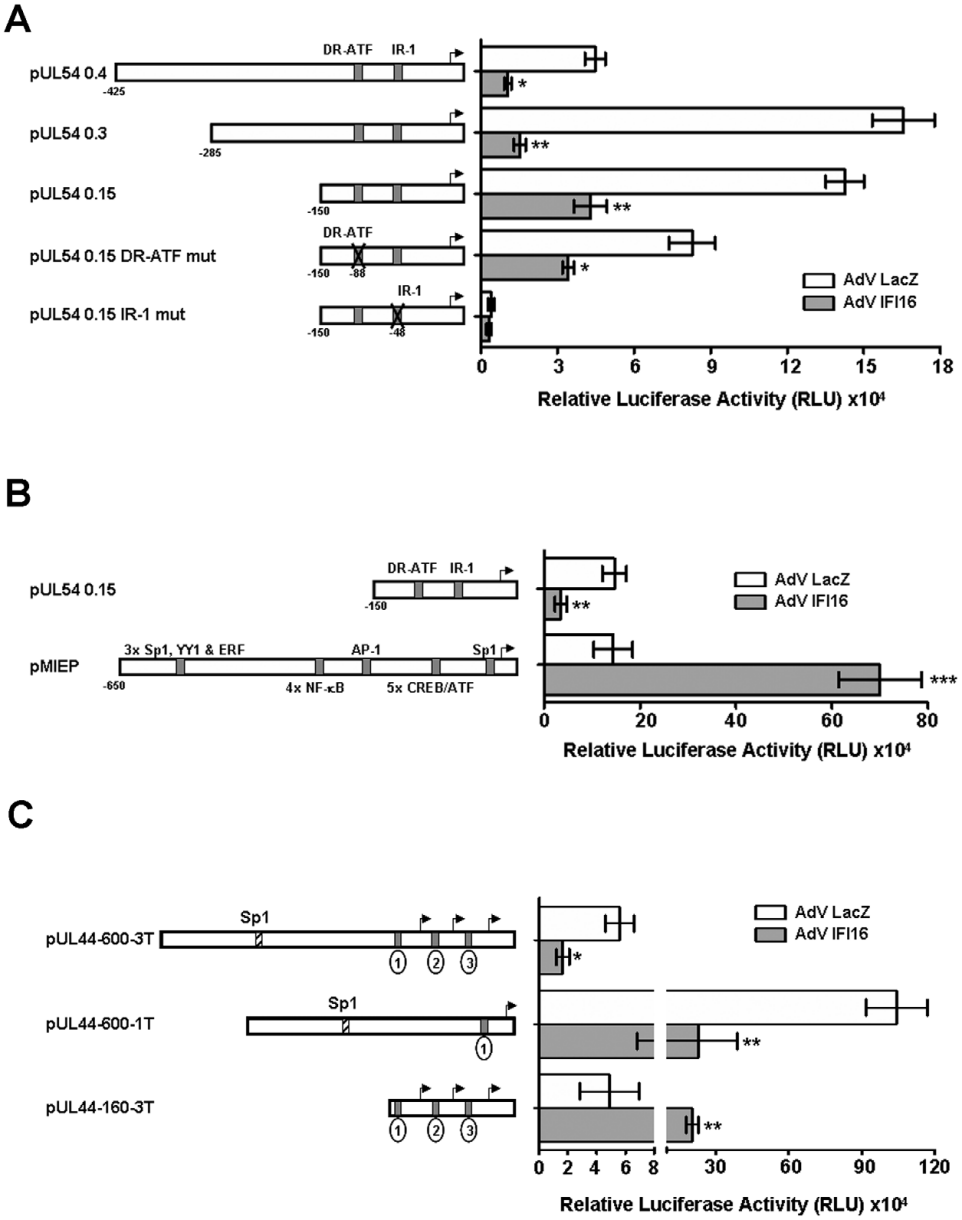
Effects of IFI16 overexpression on the activity of HCMV UL54, UL44, and MIEP promoters. A) Luciferase reporter plasmids containing HCMV UL54 promoter segments progressively deleted from the 5′ end and mutated in the IR-1 or DR-ATF elements were transfected into HELFs that were subsequently infected with either AdV LacZ or AdV IFI16 at an MOI of 200. 24 hours later, cells were infected with HCMV (MOI of 0.5) and luciferase activity assessed after a further 24 hpi. B) Luciferase reporter plasmids containing the MIEP (major immediate-early promoter**)** segment were transfected into HELFs subsequently infected as described for panel A. C) Luciferase reporter plasmids containing HCMV UL44 promoter segments progressively deleted from the 5′ end and the 3′ end were transfected into HELFs subsequently infected as described for panel A. Sp1, putative binding sites for Sp1 transcription factor; oval circles indicates TATA boxes. Experiments were repeated at least three times and one representative result is shown (mean ± SD) (*p<0.05, **p<0.01, ***p<0.001, unpaired t test for comparison of AdV LacZ *vs* AdV IFI16).

To investigate whether IFI16 overexpression may affect UL54 gene promoter activity in HCMV-infected cells, reporter plasmids containing UL54 promoter segments containing progressive 5′ deletions were transfected into HELFs left uninfected or subsequently infected with AdV IFI16 or AdV LacZ as control. Twenty-four hours later, the cells were infected with HCMV to transactivate the UL54 promoter and luciferase activity assessed following an additional 24 h. As shown in Figure 5A, HCMV infection significantly increased luciferase activity of all the reporter constructs when examined in mock- (data not shown) or LacZ-overexpressing HELFs compared to HCMV-uninfected cells. In contrast, when IFI16 was overexpressed prior to HCMV infection, luciferase activity decreased by more than 75% or 90% in cells transfected with the pUL54 0.4 or pUL54 0.3 indicator plasmids respectively, compared to AdV LacZ control cells. Similarly reduced levels (70%) were also observed with the minimal promoter construct pUL54 0.15 that contains nucleotide sequences up to −150 relative to the transcription start site, including the DR-ATF and IR-1 responsive elements. Together, these results indicate that inhibition of HCMV replication by IFI16 is consequent to DNA polymerase activity downregulation.

The HCMV DNA polymerase promoter contains a DNA element located between −54 and −43 relative to the transcription start site that has been shown to be required for both basal transcriptional activity and transactivation by IE2 [28], [29]. Mutations of the 8-bp inverted repeat element 1 (IR-1) diminishes transactivation by IE2 and abrogates the binding of cellular transcription factors, such as Sp1 [30], [31]. To investigate the involvement of IR-1 in IFI16-mediated UL54 suppression, transient transfection assays were performed using luciferase reporter constructs driven by versions of the minimal UL54 promoter (pUL54 0.15) mutated in either the DR-ATF or IR-1 element. Mutations in the DR-ATF element of the UL54 promoter (positions -82- 95) (pUL54 0.15 mut DR-ATF) slightly affected transactivation of the promoter by HCMV (141660 RLU vs. 88331 RLU) but did not impact on IFI16-mediated suppression (42930 RLU vs 34180 RLU), suggesting that the target of IFI16 might reside in the IR-1 element (Figure 5A). In accordance with the results previously reported by Luu et al. [30] and Wu et al. [31], the IR-1 mutation in the construct pUL54 0.15 (pUL54 0.15 IR-1 mut) caused more than a 95% decrease in HCMV-induced UL54 promoter activity compared to the wild type pUL54 0.15, consistent with its prominent role in the control of UL54 promoter transactivation by HCMV. IFI16 overexpression before HCMV infection did not further reduce the HCMV-driven transactivation of the IR-1 mutant construct in terms of luciferase activity, indicating that the DNA target of IFI16 suppressor activity might reside in the IR-1 element.

Suppression of UL54 promoter transactivation by IFI16 is apparently at variance with the findings of Cristea et al. [23] who showed that HCMV pUL83 stimulates activity of the major immediate-early promoter (MIEP) through its interaction with cellular IFI16 protein. Therefore, it would seem possible that positive or negative modulation of promoter activity by IFI16 might depend on the type of promoter itself and the specific transcription factors interacting. To investigate this hypothesis, the pUL54 0.15 construct and the pMIEP-Gl3 plasmid, a reporter plasmid in which the luciferase gene is driven by the HCMV major immediate-early promoter (MIEP), were transfected into HELFs that were then infected with either AdV IFI16 or AdV LacZ followed by HCMV infection. As expected, luciferase expression driven by the pUL54 0.15 promoter was inhibited (Figure 5B). In contrast, IFI16 overexpression significantly increased MIEP activity, confirming that modulation of transcription by IFI16 is highly specific and largely dependent on the type of target promoter (e.g. UL54 promoter vs. the MIEP).

To investigate whether the IFI16/Sp1 interaction affects the expression of other viral genes, the expression of UL44, a component of the HCMV DNA polymerase complex, was also investigated. UL44 protein expression was affected by IFI16 overexpression (Figure 4B), thus strengthening the evidence indicating an antiviral role of IFI16. Transfection experiments were performed using constructs containing the luciferase gene driven by the UL44 promoter containing progressive deletions in its DNA element responsive to different transcription factors [32]–[34]. Consistent with the results obtained with the UL54 promoter, IFI16 overexpression significantly reduced luciferase activity by more than 70% in cells transfected with the pUL44-600-3T plasmid (containing the UL44 promoter from −613 nt to +67 relative to the proximal transcription start site and all three TATA elements) or the pUL44-600-1T indicator plasmid (containing the UL44 promoter from −613 nt to −92 relative to the proximal transcription start site and only the distal TATA element), compared to AdV LacZ control cells. Interestingly, in the absence of the Sp1 responsive element upstream of the three transcription starting sites (from −613 nt to −164 relative to the proximal transcription start site), IFI16 stimulated the activity of the pUL44-160-3T indicator plasmid, as seen with the MIEP promoter. These results suggest that the IFI16/Sp1 interaction is important for modulating the activity of target viral genes.

**Figure 6:**
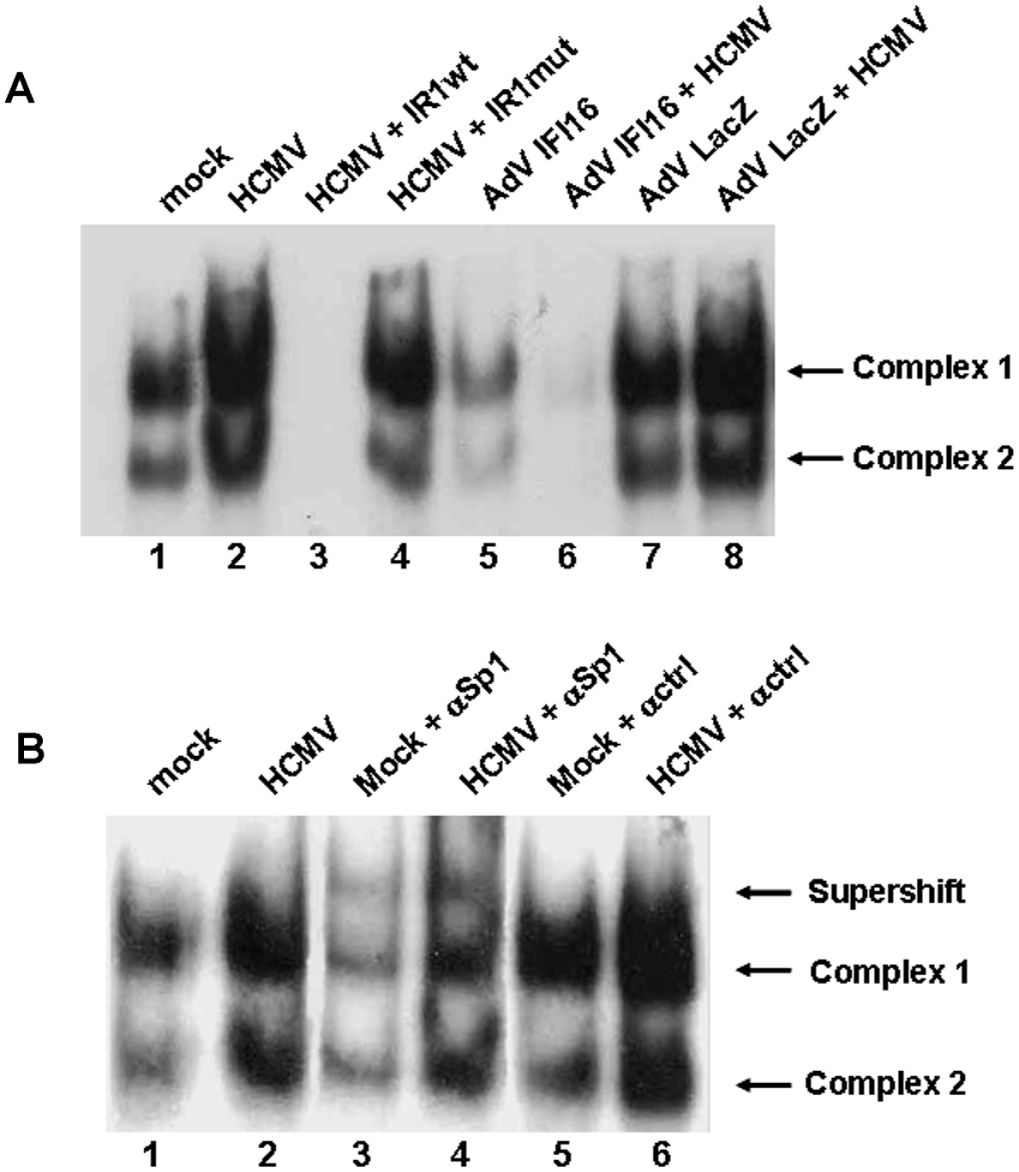
IFI16 impairs IR-1 binding. A) Nuclear protein extracts from HELFs infected with AdV IFI16 or AdV LacZ at an MOI of 200 for 24 hours and then with HCMV at an MOI of 2 for 24 hours were incubated with a radiolabeled oligonucleotide containing the consensus IR-1 binding site. Competition experiments were performed with 100-fold excess of cold specific oligonucleotide in either the wild type or the mutated form. B) Super-shift experiments were performed by adding polyclonal antibodies against Sp1, control antobody (ctrl). Experiments were repeated at least three times and one representative result is shown.

Previous mutagenesis scanning and EMSA analyses of the UL54 −54/−43 sequence (IR-1 element) indicated that Sp1 is the cellular factor responsible for abetting the action of HCMV IE proteins at the UL54 promoter [30]. To investigate whether Sp1 could be the target of IFI16, causing the modulation of IR-1 activity in the context of HCMV infection, nuclear extracts from AdV IFI16- or AdV LacZ-transduced HELFs infected at an MOI of 200 for 24 h and then with HCMV at an MOI of 2 for 24 h were analyzed by EMSA using an IR-1 oligonucleotide probe. Consistent with the results of Luu and Flores [30], the oligonucleotide spanning the IR-1 element formed two major complexes (1 and 2) with nuclear extracts from cells infected with HCMV 24 h earlier (Figure 6A, lane 2) that were reduced in the mock-infected cells (lane 1). These complexes could be specifically competed by a 100-fold excess of unlabeled IR-1wt (lane 3), but not by the same concentration of a mutated oligonucleotide (lane 4). In HELFs overexpressing IFI16 (lane 5), subsequently infected with HCMV (lane 6), and incubated with the labeled IR-1wt oligonucleotide, the generation of both complex 1 and 2 was significantly reduced at 24 hpi, suggesting that IFI16 inhibits the binding of both cellular and viral-induced transcription factors to the IR-1 sequence. The suppression of complex 1 and 2 must be due to IFI16 since infection of HELFs with AdV LacZ (used as control) does not impair the induction of the two complexes by HCMV (lanes 7 and 8). To confirm that the complexes 1 and 2 impaired by IFI16 overexpression contained Sp1, antibodies specific for Sp1 protein or unrelated antibodies (ctrl) were added to EMSA reactions for supershift analysis. As shown in Figure 6B, addition of anti-Sp1 (lanes 3 and 4), but not unrelated antibodies (lanes 5 and 6), supershifted the protein complexes of both mock and HCMV-infected cells, indicating that Sp1 is a component in both IFI16-suppressed complexes 1 and 2. In line with the results reported by Luu and Flores [30], anti-IFI16 antibodies did not alter the mobility of the two complexes, demonstrating that IFI16 does not form part of either protein complex 1 or 2 (data not shown). Altogether, these results demonstrate that suppression of UL54 promoter activity by IFI16 is associated with inhibition of the formation of Sp1-containing complexes with the IR-1 element.

### Transactivation of the UL54 promoter by HCMV is inhibited upon IFI16 binding to Sp1

**Figure 7:**
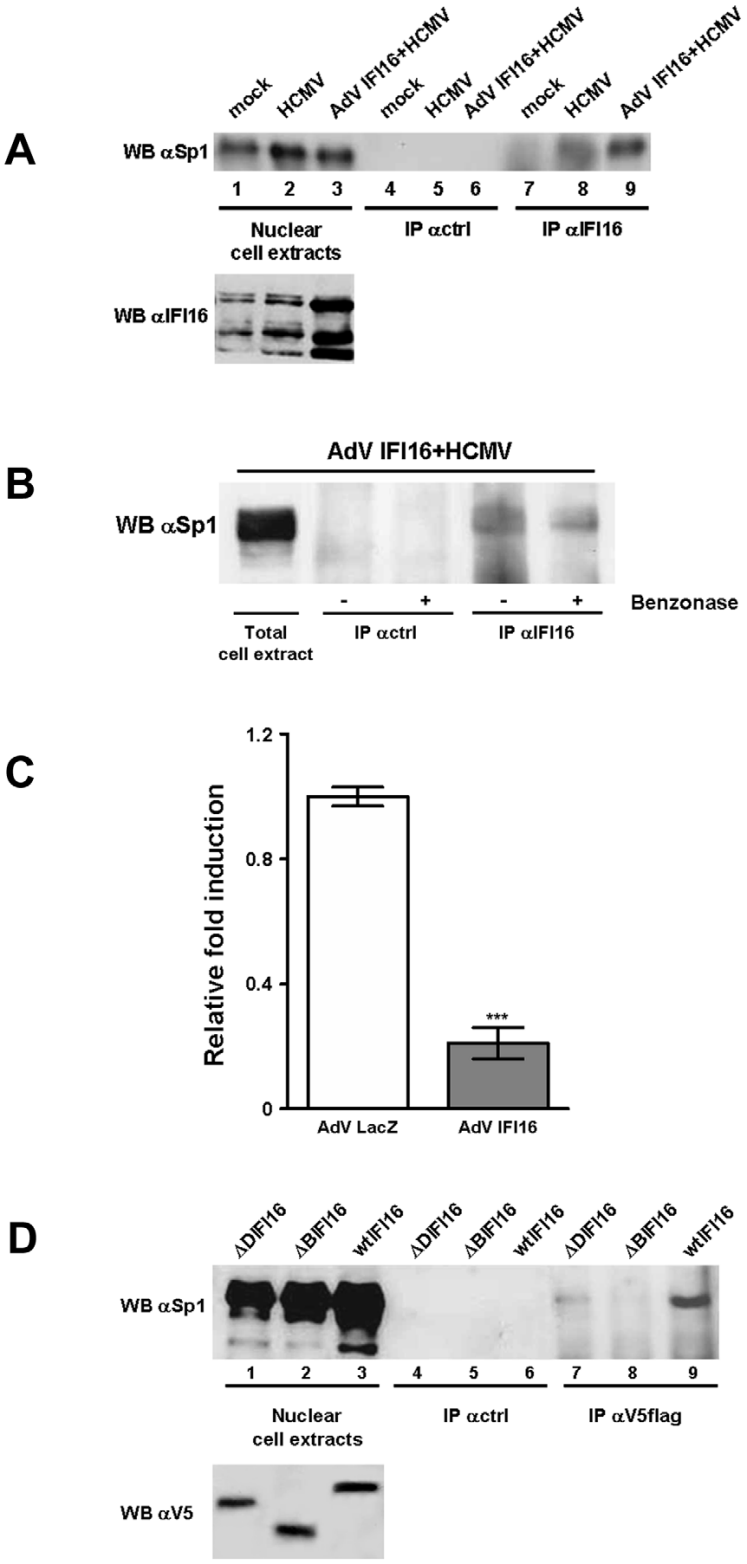
Interplay between Sp1 and IFI16 down-regulates HCMV replication. A) Nuclear cell protein extracts from HELFs mock infected (lanes 1, 4, 7), infected with HCMV at an MOI of 2 for 24 hours (lanes 2, 5, 8), or infected with AdV IFI16 or AdV LacZ (data not shown) at an MOI of 200 for 24 hours and then with HCMV (lanes 3, 6, 9) were immunoprecipitated with polyclonal antibodies against IFI16 or control antibody (ctrl). Samples were then immunoblotted with polyclonal antibodies against Sp1 or IFI16. B) Infected cell lysates were immunoprecipitated with anti-IFI16 polyclonal antibodies in the absence (lane 2 or 4) or presence of benzonase (lanes 3 and 5) or with control antibody (ctrl). Proteins from the IP were analyzed by Western blotting using polyclonal antibody recognizing Sp1. C) Nuclear extracts from HELFs mock infected or infected with AdV IFI16 or AdV LacZ at an MOI of 200 for 24 hours and then with HCMV at an MOI of 2 for 24 hours were analyzed by ChIP assay using anti-Sp1 rabbit polyclonal antibody. Sp1-coprecipitating DNA was analyzed by quantitative real-time PCR with IR-1 sequence specific primers. An unrelated rabbit polyclonal anti-serum was used as control (data not shown). Non-immunoprecipitated whole cell extract (input) obtained from AdV LacZ- or AdV IFI16-infected cells was employed to normalize the IR-1 viral DNA subjected to immunoprecipitation. Experiments were repeated at least three times and one representative result is shown (mean ± SD) (***p<0.001, unpaired t test). D) Nuclear cell protein extracts from HELFs stably transduced with the recombinant Lentivirus carrying the full-length IFI16 protein (IFI16wt), or dnIFI16 ORFs (ΔDIFI16 or ΔBIFI16), were infected with HCMV at an MOI of 2 for 24 hours, immunoprecipitated with monoclonal antibodies against flag V5 or control antibodies (ctrl), and immunoblotted with polyclonal antibodies against Sp1 (top panel) or flag V5 (bottom panel).

To identify the mechanisms underlying the suppression of Sp1-induced transcription of the UL54 promoter by IFI16, immunoprecipitation (IP) experiments and *in vivo* chromatin IP (ChIP) assays were performed. IP was carried out on infected and uninfected cell lysates using polyclonal Abs recognizing IFI16 or a control antibodies (ctrl). Immunoprecipitated proteins were examined by Western blotting using antibodies recognizing Sp1 (Figure 7A). A species of the same size as Sp1 that reacted with the polyclonal anti-Sp1 antibody could be observed in the nuclear protein extracts (Figure 7A, lanes 1, 2, 3) or and in the proteins immunoprecipitated from the infected lysates using the anti-IFI16 Abs (Figure 7A, lanes 8, 9). It is also worth noting that a band of greater intensity was observed in lysates derived from HCMV-infected cells overexpressing IFI16 (Fig. 7A, lane 9). In the uninfected cell lysates (mock), no migrating band of the same size as Sp1 could be detected (Figure 7A, lane 7). Similarly, no bands were detected when infected cell lysates were immunoprecipitated with control antibodies (ctrl) and reacted with anti-Sp1 antibodies (Figure 7A, lanes 4–6). Finally, the presence of IFI16 protein was also confirmed in the very same nuclear cell extracts (Fig. 7A, bottom panel). Thus, these results indicate that in IFI16-overexpressing and HCMV infected HELFs, IFI16 and Sp1 physically interact. It has been demonstrated elsewhere that IFI16 binds to DNA [35]. DNA-binding proteins can associate during IP due to their adjacent binding on DNA rather that due to protein-protein interactions [36]. To determine whether nucleic acid is required for the IFI16/Sp1 association, IP was performed using an anti-IFI16 Ab or a control Ab (ctrl) in the presence or absence of benzonase (a non specific nuclease), and the IPs were probed by Western blot using anti-Sp1 Ab. No Sp1 was detected in IPs using the control Ab (Figure 7B, lanes 2 and 3). In contrast, Sp1 was detected in IPs from infected lysates in both the absence (lane 4) and presence (lane 5) of benzonase. To confirm the action of benzonase on nucleic acid, the cell lysates analyzed in Figure 7B were examined on an ethidium bromide-stained agarose gel. In the absence of benzonase, a robust staining of nucleic acid could be seen, whereas staining in the presence of benzonase was no more intense than that of a no-sample control (data not shown). Altogether, these results demonstrate that suppression of UL54 transcription by IFI16 is accompanied by the displacement of Sp1 from its promoter due to its direct association with IFI16.

To corroborate these results further, the binding of Sp1 to the UL54 promoter in the presence of IFI16 was then analyzed *in vivo* by ChIP assay. Formaldehyde cross-linked sonicated chromatin fragments from HELFs infected with AdV IFI16 or AdV LacZ 24 h earlier and then infected with HCMV for 24 h were immunoprecipitated using an anti-Sp1 polyclonal antibody. The DNA released from the immunocomplexes was then analyzed by quantitative real-time PCR to detect the enrichment of the IR-1 sequence of the UL54 promoter in the immunoprecipitates. The rate of amplification was verified using cross-linked non-immunoprecipitated chromatin (input). Consistent with the EMSA and IP results, real-time PCR analysis of the purified ChIPed DNA showed that the Sp1 antibody pulled approximately 80% more Sp1-bound UL54 promoter DNA down in extracts from AdV LacZ-infected HELFs compared with those from AdV IFI16-infected HELFs (Figure 7C). Unrelated, affinity-purified polyclonal antibodies, used as negative control, did not immunoprecipitate the complex containing the UL54 promoter (data not shown). Altogether, these findings indicate that the suppression of UL54 gene transcription in the presence of IFI16 is due to the inhibition of Sp1 recruitment to its promoter.

To learn more about the basis of the IFI16/Sp1 interaction and its effects on HCMV replication, cell lines stably expressing the V5-tagged mutant forms of IFI16, namely ΔDIFI16, ΔBIFI16, or the full-length IFI16 (wtIFI16), were infected with HCMV at an MOI of 2 PFU/cell. Twenty-four hours later, IP was carried out using monoclonal Ab recognizing the V5 tag, or control antibody (ctrl). Immunoprecipitated proteins were examined by Western blotting using antibodies recognizing Sp1. As shown in Figure 7D, a species of the same size as Sp1 could be observed in the total nuclear extracts (Figure 7D, top panel, lane 1–3). A band migrating at a similar molecular weight could also be observed in the proteins immunoprecipitated from HELFs overexpressing the full length IFI16 form (wtIFI16) (Figure 7D, lane 9). In contrast, no bands corresponding to Sp1 could be observed in the immunoprecipitates from the ΔBIFI16 cell line when blotted with anti-Sp1 antibodies (Figure 7D, lane 8). A detectable, although weaker band corresponding to Sp1 could be observed in the immunoprecipitates from the ΔDIFI16 cell line (Fig. 7D, lane 7). As expected, protein extracts immunoprecipitated from infected cell lines with control antibodies (ctrl) did not display any migrating band (Figure 7D, lane 4–6). Finally, the presence of V5-tagged proteins was also confirmed in the very same nuclear cell extracts (Fig. 7D, bottom panel). Altogether, these results demonstrate that the interaction of IFI16 with Sp1 most likely depends on the integrity of the HIN domains present on the full length protein. In support of this hypothesis, the ΔDIFI16 mutant containing the two HIN domains partially maintained its ability to bind Sp1, although at levels much lower than those of the wtIFI16. By contrast, the ΔBIFI16 mutant lacking the HIN-B domain completely lost its ability to interact with Sp1.

### Suppression of HCMV replication by IFI16 does not require IFN-β antiviral activity

**Figure 8:**
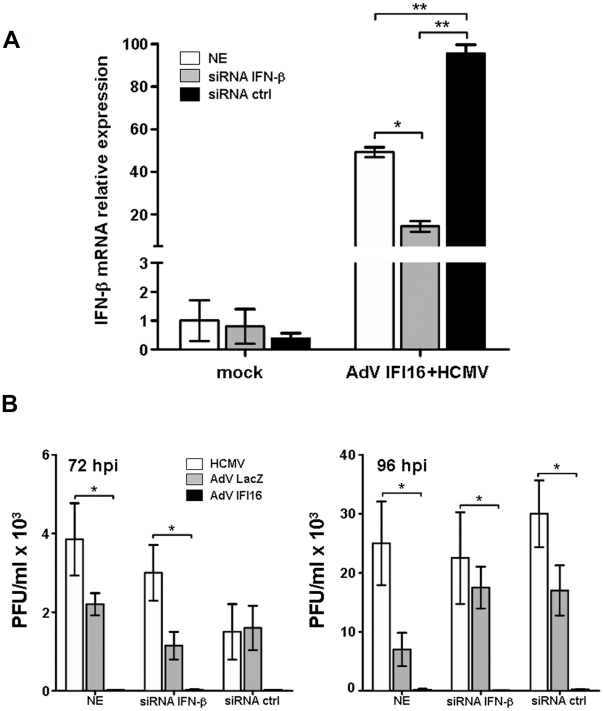
Suppression of HCMV replication by IFI16 does not require IFN-β antiviral activity. HELFs were electroporated with a mixture of four different small interfering RNA (siRNA IFN-β) or scrambled control siRNA (siRNA ctrl), or left not electroporated (NE), and then infected with AdV IFI16 or AdV LacZ (MOI of 200 PFU/cell) before subsequent infection with HCMV 24 hours later (MOI of 0.1 PFU/cell). A) Total RNA was isolated 24 hours post HCMV infection and IFN-β mRNA expression was determined by quantitative real-time PCR. Levels of IFN-β mRNA are presented normalized to the levels of cellular β-actin. Experiments were repeated at least three times and one representative result is shown (mean ± SD). B) Cell-free supernatants were harvested at the indicated hours post infection (hpi) and virus amounts determined by plaque assay. The data shown are the average of three experiments ± SD (*p<0.05, **p<0.01, one-way ANOVA followed by Bonferroni's post test).

IFI16 was recently identified to be an intracellular sensor of HSV-1 DNA, which stimulates the expression of IFN-β and pro-inflammatory genes through activation of IRF3 and NF-κB transcription factors [8]. To clarify whether IFI16 could suppress HCMV replication through IFN-β induction, HELFs were electroporated with siRNA specific for IFN-β or siRNA ctrl or left not electroporated (NE), infected with AdV IFI16 at an MOI of 200, and 24 h later with HCMV at an MOI of 0.1. At different time points post infection, HCMV yield was measured. As shown in Figure 8A, this treatment led to the inhibition of IFN-β induction in response to HCMV infection. By contrast, suppression of IFN-β production did not prevent the inhibition of HCMV replication by IFI16 overexpression, demonstrating that IFI16 does not require functional IFN-β and that it directly inhibits HCMV replication (Figure 8B). IFN-β production was wiped out by siRNA specific for IFN-β, as proven by the finding that an inducer of IFN-β (poly I:C) failed to block HCMV replication upon HELF treatment with siRNA IFN-β, while it was blocked in cells treated with siRNA ctrl (data not shown).

## Discussion

This study demonstrates that IFI16 acts as restriction factor for HCMV replication. Inhibition of HCMV growth was observed by adopting two different experimental approaches. In the first approach, primary HELFs were generated whereby the IFI16 protein underwent a long-lasting knockdown through the use of either siRNAs or the overexpression of dominant negative forms of the IFI16 protein that lacked the death domain (PYD) at the N-terminus or the HIN-B domain at the C-terminus. HELFs were chosen for two main reasons: i) they are fully permissive to HCMV; and ii) they are characterized by a very low basal level of IFI16 expression that can be adequately up-regulated following transduction with the AdV IFI16 vector. When HCMV replication was analyzed in these cells, in either the absence of IFI16 or in the presence of an inactivated form of IFI16, viral yield at low MOI was significantly increased. The finding that IFI16 counteracts HCMV replication is further supported by the outcome of the experiments using HELFs overexpressing IFI16. In these cell cultures, the HCMV yield at low MOI was severely impaired, confirming that IFI16 is indeed endowed with antiviral activity. Viral gene expression analysis on both the mRNA and protein level showed that IFI16 did not affect IE expression, but rather a viral replication step down-stream of IE expression. In line with this finding, and by measuring the DNA viral load, we demonstrated that IFI16 down-regulates viral DNA synthesis by affecting the *bona fide* activity of the UL54 DNA polymerase gene and the UL44 gene. Definitive support for this came from transfection experiments which showed IFI16 overexpression to significantly impair the activity of both UL54 and UL44 gene promoters, responsible for viral DNA synthesis. Previous studies have indeed shown that IFI16 can be purified by DNA affinity chromatography using a region of the UL54 [30]. Moreover, cotransfection of IFI16 and a CAT reporter gene containing the wild type UL54 promoter results in a dose-dependent decrease in reporter activity [27], suggesting an interplay between IFI16 and transcription factors responsible for UL54 promoter activation. All these observations were made, however, in uninfected cells using the UL54 essential promoter as a simple target of cellular transcription factors and the mechanisms responsible for the inhibition of UL54 transactivation by IFI16 in infected cells remained to be elucidated. Therefore, to provide new evidence about the action of IFI16 in the context of HCMV infection, promoter scan analyses, EMSA and ChIP, were each performed in IFI16-overexpressing HELFs infected with HCMV. The results obtained demonstrate that in order to exert its antiviral activity IFI16 binds and displaces the Sp1 transcription factor interacting with the responsive IR-1 element present in the UL54 promoter. Sp1 detachment from its DNA cognate element leads to a decrease in HCMV DNA synthesis and, as a consequence, the inhibition of virus replication. Consistent with this, we have previously demonstrated that activation of the NF-κB response is mediated by an IFI16-induced blockade of Sp1-like factor recruitment to the promoter of the IκBα gene, which encodes the main NF-κB inhibitor [37].

In order to provide new insights into the mechanisms of the interaction of IFI16 with Sp1 and its effects on HCMV replication, we produced cell lines overexpressing mutated forms of IFI16 lacking the HIN-B domain (ΔBIFI16) or the PYD domain (ΔDIFI16). Virus yield experiments demonstrated that HCMV replication in the ΔDIFI16 cell line was enhanced compared to that in the cell lines expressing the wild type IFI16 or IFI16 lacking the HIN-B domain (ΔBIFI16). A conceivable explanation for these results could be the following. The mutated form of IFI16 lacking the HIN-B domain (ΔBIFI16) is unable to physically interact with Sp1 and therefore can no longer relieve the suppressive activity of endogenous IFI16 on viral promoters, such as UL54 and UL44. In contrast, in cells lacking the PYD domain (ΔDIFI16), IFI16 to some extent maintains its capability to interact with Sp1 and compete with the endogenous form on the viral promoter. As a consequence, the suppressive activity of the endogenous IFI16 protein is retained, stimulating HCMV replication. The finding that the HIN-B domain is responsible for the Sp1 interaction is in line with results recently reported by Liao et al. [38], which show that the HIN-B and HIN-A domains are together responsible for the IFI16/p53 interaction. These results corroborate the notion that IFI16 is a modular protein and that its different functions correspond to its different domains.

A different role of IFI16 in HCMV replication has been demonstrated by Cristea et al. [23], who identified the interaction of pUL83 (pp65) with IFI16 throughout the course of HCMV infection and showed that pUL83 recruits IFI16 to the major immediate-early promoter (MIEP) and stimulates, rather than inhibiting, MIEP activity. Consistent with these observations, when we compared UL54 promoter activity with MIEP activity in IFI16 overexpressing cells, stimulation of only the latter promoter was observed. The differential sensitivity of the two promoters to IFI16 activity may be explained by the presence of four NF-κB responsive elements on the MIEP. Functional analysis of the ICAM-1 promoter by deletion- or site-specific mutagenesis has indeed demonstrated that NF-κB is the main mediator of IFI16-driven gene induction [37]. NF-κB activation appears to be triggered by IFI16 through a novel mechanism involving suppression of IκBα mRNA and protein expression. Furthermore, to study the activity of IFI16 in p53-mediated gene expression, Fujiuchi et al. [39] examined BAX promoter (a p53 target gene) activation by coexpressing p53 and IFI16. When the proteins were coexpressed, promoter activity was enhanced up to 17-fold. Consistent with the results showing the collaboration of p53 and IFI16 in transcription, endogenous levels of BAX, p21WAF1, and HDM2 were synergistically induced by expressing both proteins, as shown by Western blot analysis. Taken together, these results demonstrate that depending on the factors and the type of promoter it is interacting with, IFI16 may act either as a positive or negative transcription regulatory factor. Moreover, we have previously observed that murine CMV (MCMV) replication was delayed in mouse embryo fibroblasts (MEF) following inactivation of the IFI16 mouse homolog Ifi204 with a p204-dominant-negative mutant. These results suggested that the activity of this protein was required for efficient MCMV replication [24]. These discrepancies may be thus explained by the findings that IFI16 requirement varies during HCMV replication and depends on the MOI employed.

IFI16 has been shown to act as an innate immune sensor of intracellular dsDNA [35]. Upon sensing dsDNA, the IFI16 protein triggers the induction of IFN-β. IFI16 directly associated with IFN-β-inducing viral DNA motifs recruits STING a critical mediator of the IFN-β response to DNA. It is therefore possible that IFI16 inhibits HCMV replication through the induction of IFN-β. This is quite unlikely, however, in the light of the following observations. First of all, the outcome of the transfection experiments and EMSA indicates that IFI16 directly interacts with and down-regulates Sp1, which is responsible for UL54 promoter activation. Secondly, knockdown of the IFN-β gene by specific siRNAs does not impair the ability of IFI16 to down-regulate HCMV replication. Finally, the addition of anti-IFN-type I antibodies does not impair the capability of IFI16 to suppress HCMV replication (unpublished). Thus, IFI16 appears to directly inhibit virus HCMV replication rather than down-regulating viral growth through activation of an IFN pathway.

Overall, although the detailed mechanisms of IFI16-mediated repression of viral E and L gene expression remain to be fully determined, the results presented in this study congruently demonstrate that the actions of IFI16 contribute to a cell's intrinsic repression mechanism of HCMV gene expression. It remains to be determined, however, how the virus counteracts IFI16 activity and shifts the balance toward viral evasion and its consequent growth.

## Materials and Methods

### Cells and viruses

Low-passage human embryonic lung fibroblasts (HELFs), human embryo kidney 293 cells (HEK 293) (Microbix Biosystems Inc.), African green monkey kidney cells (Vero) and mouse connective tissue fibroblasts (L929) were grown in Eagle's minimal essential medium (Gibco-BRL) supplemented with 10% fetal bovine serum (FBS; Gibco-BRL). Human umbilical vein endothelial cells (HUVECs) were isolated by trypsin treatment of umbilical cord veins cultured in Endothelial Growth Medium (EGM) corresponding to Endothelial Basal Medium (EBM) (Clonetics, San Diego, CA) containing 2% FCS, human recombinant vascular endothelial growth factor (hrVEGF), basic fibroblast growth factor (bFGF), human epidermal growth factor (hEGF), insulin growth factor (IGF-1), hydrocortisone, ascorbic acid, heparin, gentamycin and amphotericin B (1 mg/ml each). Experiments were carried out with cells at passages 4–8. HCMV strain AD169 (ATCC-VR538) and a clinical isolate of Adenovirus were propagated on HELF cells, clinical isolates of HSV-1 and HSV-2 on Vero cells and Vesicular Stomatitis Virus (VSV) serotype Indiana on L929 cells and titrated by standard plaque assay, as previously described [40]. HCMV VR1814 is a derivative of a clinical isolate and grows efficiently on HUVECs [41].

### Inhibition of IFI16 and IFN-β expression

HELF cells were transiently transfected with a MicroPorator (Digital Bio) according to the manufacturer's instructions (1200 V, 30 ms pulse width, one impulse) with a pool of IFI16 small interfering RNAs (siRNA IFI16), siRNA IFN-β, or control siRNA (siRNA ctrl) as negative control (final concentration: 300 nM; Qiagen). The IFI16 and IFN-β siRNA sequences are reported in Table S1. IFI16 or IFN-β siRNA-induced blockade was checked by immunoblotting with rabbit anti-IFI16 antibodies or by real-time PCR with IFN-β specific primers (Table S1) respectively, at the time points indicated.

### Recombinant lentiviral and adenoviral vectors

Lentiviral vectors carrying the full-length IFI16 ORF (wt IFI16) or IFI16 ORF lacking the PYD domain (ΔPYDIFI16) or the HIN-B domain (ΔHIN-BIFI16) or the LacZ gene as a control, were generated as described by Azzimonti et al. [42]. To obtain lentiviral lines, HELFs were transduced with the recombinant Lentivirus and successfully transduced cells selected using blasticidin (4 µg/ml; for a maximum of 10 days). Transduction efficiency was assessed by immunoblotting for the V5-epitope. The adenovirus transfer vector pAC-CMV IFI16 was constructed as described in Gugliesi et al. [43].

### Plasmids

The HCMV UL54 (pol) promoter sequences (positions −425 to +15 relative to the UL54 transcription start site, GenBank NC_006273) were amplified by PCR using purified HCMV AD169 DNA as the template and the primer sets reported in Table S1. The 5′- and 3′-primers were engineered with HindIII and KpnI restriction sites. The PCR fragments were subsequently digested and directionally cloned into the corresponding sites of the pGL3-basic vector (Promega) to obtain the pUL54 0.4 construct. The pUL54 0.3 and pUL54 0.15 constructs were derived from the pUL54 0.4 construct and contain UL54 promoter sequences −285 to +15 and −150 to +15, respectively. These constructs were generated by PCR using the UL54 appropriate primers (Table S1). The fragments were then ligated into the HindIII and KpnI sites of the pGL3-basic vector. To obtain UL54-0.15 promoter sequence with inactivated IR-1 and DR-ATF binding sites, the sequences of these sites were modified by site-directed mutagenesis (Quick Change XL Site-Direct Mutagenesis Kit, Stratagene). The IR-1 (−54 to −42) and DR-ATF (−97 to −79) recognition sites of pUL54 0.15 were changed into unique restriction sites (−41 to −47, XbaI, and −89 to −95, EcoRI, respectively) using the IR-1 mutant and DR-ATF mutant oligonucleotides (Table S1) and their complementary oligonucleotides. The HCMV IE promoter-enhancer sequence (position −666 to +19 relative to the IE1/IE2 transcription start site, GenBank K03104.1) was amplified out of the purified HCMV AD169 genome by PCR using the primer sets shown in Table S1. The 5′- and 3′-primers were engineered using NheI and HindIII restriction sites (underlined). The PCR fragments were subsequently digested and directionally cloned into the corresponding sites of the pGL3-basic vector (Promega) to obtain the pMIEP construct. The correctness of all the amplified viral sequences was confirmed by sequencing. The HCMV UL44 promoter sequences (positions −613 to +67 relative to the proximal UL44 transcription start site, GenBank NC_006273) were amplified by PCR using purified HCMV AD169 DNA as the template and the primer sets reported in Table S1. The 5′- and 3′-primers were engineered with HindIII and BglII restriction sites. The PCR fragments were subsequently digested and directionally cloned into the corresponding sites of the pGL3-basic vector (Promega) to obtain the pUL44-600-3T construct. The pUL44-660-1T and pUL44-160-3T constructs were derived from the pUL44-600-3T construct and contain the UL44 promoter sequences −613 to −92 and −164 to +67 relative to the proximal UL44 transcription start site, respectively. These constructs were generated by PCR using the UL44 appropriate primers (Table S1). The fragments were then ligated into the HindIII and BglII sites of the pGL3-basic vector. The correctness of all the amplified viral sequences was confirmed by sequencing.

### Transfections and luciferase assay

Cells were electroporated using a Micro-Porator MP-100 (Digital BioTechnology), according to the manufacturer's instructions (a single 1400 V pulse, 20 ms pulse width). After 24 h, cells were infected with AdV IFI16 or the control indicator plasmid AdV LacZ (MOI of 200 PFU/ml) and 24 h later the cells were infected with HCMV AD169 (MOI of 0.5). Following a further 24 h, luciferase activity was measured using the Dual Luciferase Reporter Assay System Kit (Promega) on a Lumino luminometer (Stratec Biomedical Systems, Birkenfeld, Germany), as previously described by Baggetta et al. [44].

### Immunoblotting

Whole-cell protein extracts were prepared and subjected to immunoblot analysis as described in Gugliesi et al.[43]. The following antibodies were used: rabbit polyclonal anti- C-terminal IFI16 antibodies (diluted 1∶1000) or mouse monoclonal antibodies (MAb) anti- IEA (IE1 plus IE2, 11-003; Argene, diluted 1∶250), UL44 (P1202-2, Virusys, clone CH16, diluted 1∶500), UL83 (CA003-100, Virusys, clone 3A12, diluted 1∶1000), V5 (R960-25, Invitrogen, diluted 1∶5000); MAb against β-actin (MAB1501R; Chemicon, Temecula, CA, diluted 1∶2000) were used as a control for protein loading. Immunocomplexes were detected with sheep anti-mouse or donkey anti-rabbit immunoglobulin antibodies conjugated to horseradish peroxidase (Amersham) and visualized by enhanced chemiluminescence (Super Signal; Pierce).

### Nuclear extract isolation and electrophoretic mobility shift assay (EMSA)

HELFs were infected with AdV IFI16 or control plasmid AdV LacZ (MOI of 200 PFU/cell) for 24 h and subsequently infected with HCMV strain AD169 (MOI of 2 PFU/cell) for 24 h. Nuclear extracts were collected using the Nuclear Extract Kit (Active Motif) according to the manufacturer's instructions. Electrophoretic mobility shift assays were carried out as previously described [37]. Briefly, nuclear extracts (15 µg of protein) were incubated in a binding buffer (10 mM Tris-HCl [pH 7.5], 50 mM NaCl, 0.5 mM EDTA, 0.5 mM dithiothreitol, 1 mM MgCl2, 5% glycerol) containing 2 µg of poly (dI-dC) (GE Healthcare) and the ^32^P oligonucleotide probe representing the wild-type (wt) or mutated (mut) HCMV UL54 promoter IR-1 motif (− 65 to −35 respect to the transcription start site). Sequences are reported in Table S1. For supershift experiments, 2 µg of polyclonal antibody recognizing Sp1 (Millipore) or 2 µg of rabbit polyclonal anti-human C-terminal IFI16 antibodies were used. Unlabeled 30-bp annealed oligonucleotide was added as the competitor DNA in 100-fold molar excess above the level of the probe.

### Immunoprecipitation

For immunoprecipitation experiments, nuclear cell proteins were obtained as described for EMSA analysis. 30 µg of proteins were incubated with antibodies of interest (2 µg) for 1 h at room temperature with rotation. The immune complexes were collected using protein G–Sepharose beads (Sigma-Aldrich) for an additional 1 h at room temperature with rotation. The Sepharose beads were pelleted and washed three times with RIPA buffer. Finally, the proteins were eluted using Laemmli sample buffer and resolved on 8% SDS-PAGE gel to assess the protein binding by Western blotting. Where indicated, 400 U benzonase (Novagen) was added for 30 minutes at 4°C after clarification of the lysate by centrifugation, as described in Strang et al. [45].

### Chromatin Immunoprecipitation (ChIP) assay

The ChIP assay was performed as previously described [37]. Briefly, HELFs were infected with AdV IFI16 at an MOI of 200 for 24 h and then with HCMV at an MOI of 2 for 24h. DNA-protein complexes were cross-linked with PBS containing 1% formaldehyde for 10 min, and the reaction was stopped via the addition of glycine to a final concentration of 125 mM for 5 min at room temperature. Nuclear extracts were collected by using the nuclear Nuclear Extract Kit (Active Motif) according to the manufacturer's instructions. Nuclear extract were sonicated to shear chromatin to a final size of 500–2000 bp and the supernatant recovered and used directly for immunoprecipitation experiments by incubation with appropriate antibodies (2 µg) for 1 h at room temperature. The immune complexes were collected as described above with protein G–Sepharose beads (SIGMA). After immunoprecipitation, beads were collected and sequentially washed as described in Caposio et al. [37]. DNA-protein cross-links were reversed by incubation in 1% SDS/TE buffer at 65°C overnight. The samples were digested with proteinase K and DNA was extracted by phenol/chloroform/isoamyl alcohol and then incubated for 30 min at 37°C TE/RNase A buffer. The input lysates were processed as above. DNA was analyzed by quantitative real-time Sybr green PCR using primers for the IR-1 sequence; the primer sequences used are: forward: 5′- GGTCCTTTGCGACCAGAAT- 3′; reverse: 5′- TATACTCGACAGCGGCGTCT- 3′. The amount of the DNA precipitated by the antibody was normalized to the total input DNA that was not subjected to immunoprecipitation. The value of 1 was assigned to the normalized level of IR-1 immunoprecipitated with unrelated antibody.

### Quantitative nucleic acid analysis

Real-time quantitative reverse transcription-PCR (RT-PCR) analysis was performed on an Mx 3000 P apparatus (Stratagene). Total RNA was extracted with the NucleoSpin RNA kit (Macherey-Nagel) and 1 µg was retrotranscribed using the Revert-Aid H-Minus FirstStrand cDNA Synthesis Kit (Fermentas). Reverse-transcribed cDNAs were amplified in duplicate using Brilliant Sybr green QPCR master mix (Fermentas), as described in Luganini et al. [40] for viral genes or for cellular cytokines [44].

To determine the number of viral DNA genomes per nanogram of cellular reference DNA (18S rRNA gene), viral DNA levels were measured by quantitative real-time PCR, as described in Luganini et al. [40], using the previously reported probe and primers to amplify a segment of the IE1 gene [46]. HCMV DNA copy numbers were normalized by dividing by the amount of human 18S rRNA gene (Assay-on- Demand, 18S, assay no. HS99999901_s1; Applied Biosystems) amplified per reaction mixture. A standard curve of serially diluted genomic DNA mixed with an IE1-encoding plasmid (from 10^7^ to 1 copy) was created in parallel with each analysis [47].

### In vitro analysis of caspase activities

Caspase 3–7 protease activity was assessed by measuring the extent of cleavage of a fluorometric peptide substrate using the SensoLyte AFC Caspase Sampler Kit “Fluorimetric” (Anaspec). Doxorubicin treatment (0.5 µM for 18 hours) was used for the positive control. Experiments were performed according to the manufacturer's instructions. Fluorescence was measured at an excitation wavelength of 405 nm and an emission wavelength of 500 nm using the VICTOR^3^ 1420 multilabel counter (Perkin–Elmer). Protease activity was expressed as fold induction relative to the basal level measured in each uninfected cell line.

### Statistical analysis

All statistical tests were performed using GraphPad Prism version 5.00 for Windows (GraphPad Software, San Diego California USA, www.graphpad.com). The data were presented as the means ± standard deviations (SD). Means between two groups were compared by using a two-tailed t-test.

Means between three groups were compared by using a one-way or two-way analysis of variance with Bonferroni's post-test. Differences were considered statistically significant at p<0.05.

## Supporting Information

Figure S1 **Assessment of the biological activity of dominant negative IFI16 (dnIFI16). A)** HELFs carrying the dnIFI16 (ΔDIFI16 or ΔBIFI16) or the control LacZ gene were infected with AdV IFI16 (MOI of 50 PFU/cell). Total RNA was isolated at 24 hpi and assayed by quantitative real-time PCR to determine the relative levels of proinflammatory gene transcripts. Levels of cellular mRNA are presented normalized to the levels of β-actin. The data shown are the average of three experiments ± SD (*p<0.05, ** p<0.01, one-way ANOVA followed by Bonferroni's post test). **B)** HELFs carrying the dnIFI16 (ΔDIFI16 or ΔBIFI16), the control LacZ gene or left mock infected, were infected with AdV IFI16 (MOI of 1 to 50 PFU/cell) or treated with doxorubicin (doxo) as positive control. At 48 hpi, cells were harvested and equal amounts of cytosolic proteins subjected to a fluorogenic caspase assay to measure the extent of protease activity. The extent of cleavage of fluorometric peptide substrate was assessed, and protease activity was expressed as fold induction relative to the basal level measured in each uninfected cell line. The data shown are the average of three experiments ± SD (**p<0.01, ***p<0.001 one-way ANOVA followed by Bonferroni's post test).
(TIF)

Figure S2 **Effect of IFI16 silencing on HCMV growth in endothelial cells.** HUVECs were electroporated with a mixture of four different small interfering RNA (siRNA IFI16) or scrambled control siRNA (siRNA ctrl) or left not electroporated (NE), and then infected with VR1814 at an MOI of 1 or 0.1 PFU/cell. Cell-free supernatants were harvested 96 hours post infection (hpi) and virus amounts determined by plaque assay. The data shown are the average of three experiments ± SD (**p<0.01, ***p<0.001 one-way ANOVA followed by Bonferroni's post test).
(TIF)

Table S1 **Oligonucleotide primer sequences.**
(DOC)
