# Supplementary figures and images for: The Intracellular DNA Sensor IFI16 Gene Acts as Restriction Factor for Human Cytomegalovirus Replication
Source: PLoS Pathog. 2012 Jan 26;8(1):e1002498. doi: 10.1371/journal.ppat.1002498 (PMC3266931; doi:10.1371/journal.ppat.1002498)

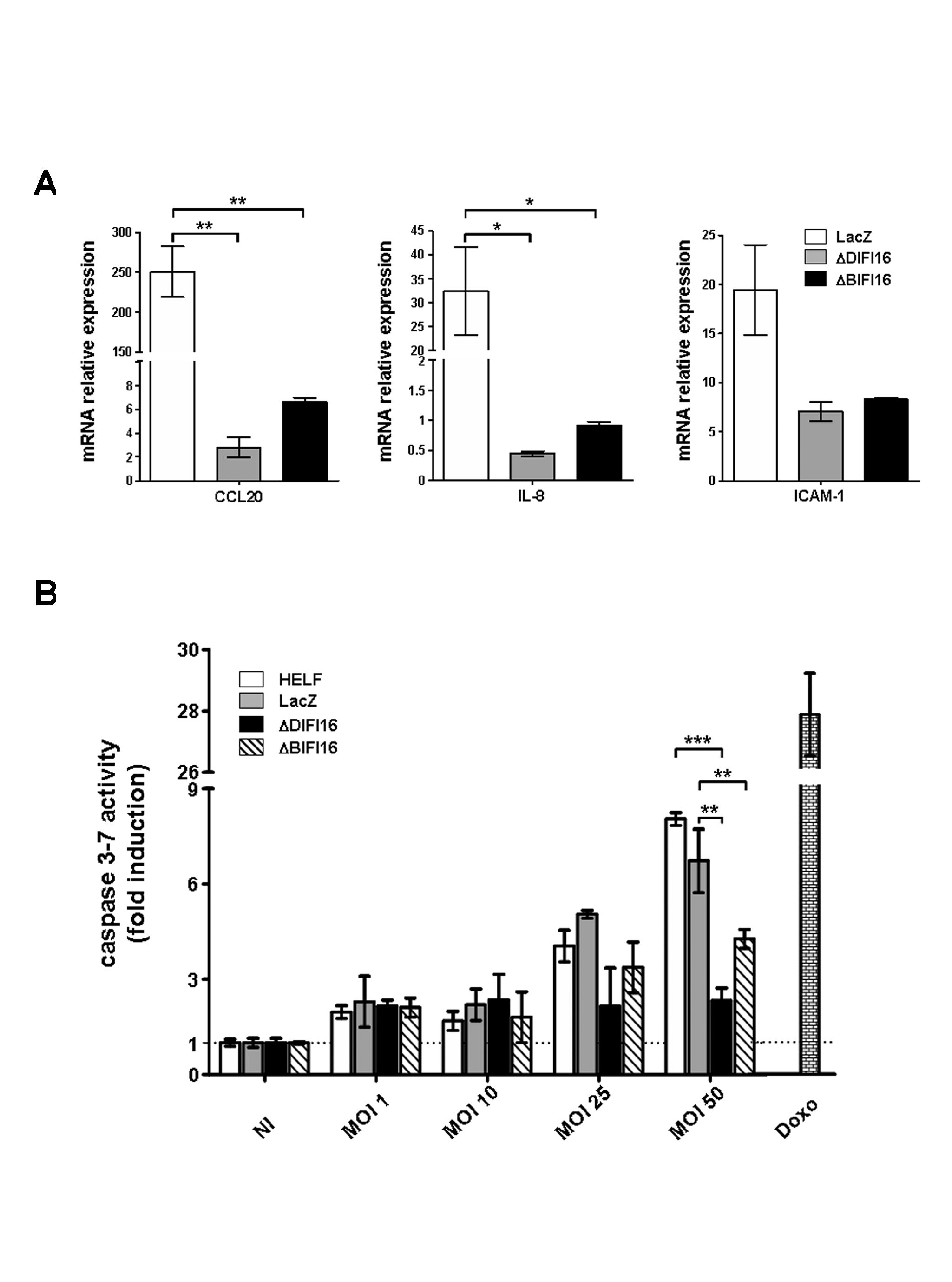

Supplement: Figure S1 — Assessment of the biological activity of dominant negative IFI16 (dnIFI16). A) HELFs carrying the dnIFI16 (ΔDIFI16 or ΔBIFI16) or the control LacZ gene were infected with AdV IFI16 (MOI of 50 PFU/cell). Total RNA was isolated at 24 hpi and assayed by quantitative real-time PCR to determine the relative levels of proinflammatory gene transcripts. Levels of cellular mRNA are presented normalized to the levels of β-actin. The data shown are the average of three experiments ± SD (*p<0.05, ** p<0.01, one-way ANOVA followed by Bonferroni's post test). B) HELFs carrying the dnIFI16 (ΔDIFI16 or ΔBIFI16), the control LacZ gene or left mock infected, were infected with AdV IFI16 (MOI of 1 to 50 PFU/cell) or treated with doxorubicin (doxo) as positive control. At 48 hpi, cells were harvested and equal amounts of cytosolic proteins subjected to a fluorogenic caspase assay to measure the extent of protease activity. The extent of cleavage of fluorometric peptide substrate was assessed, and protease activity was expressed as fold induction relative to the basal level measured in each uninfected cell line. The data shown are the average of three experiments ± SD (**p<0.01, ***p<0.001 one-way ANOVA followed by Bonferroni's post test). (TIF) [file ppat.1002498.s001.tif]

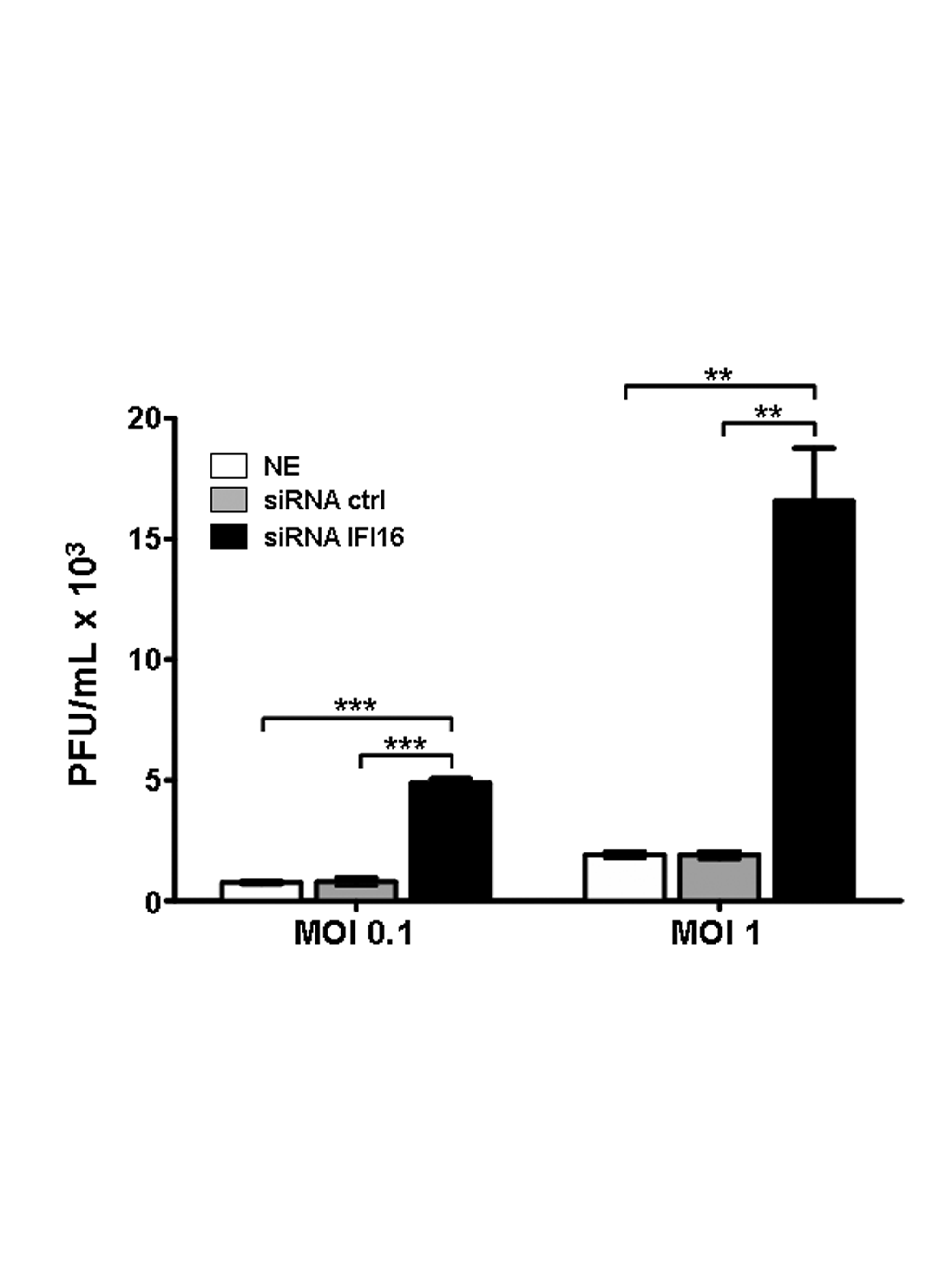

Supplement: Figure S2 — Effect of IFI16 silencing on HCMV growth in endothelial cells. HUVECs were electroporated with a mixture of four different small interfering RNA (siRNA IFI16) or scrambled control siRNA (siRNA ctrl) or left not electroporated (NE), and then infected with VR1814 at an MOI of 1 or 0.1 PFU/cell. Cell-free supernatants were harvested 96 hours post infection (hpi) and virus amounts determined by plaque assay. The data shown are the average of three experiments ± SD (**p<0.01, ***p<0.001 one-way ANOVA followed by Bonferroni's post test). (TIF) [file ppat.1002498.s002.tif]
