# Supplementary material for: The Intracellular DNA Sensor IFI16 Gene Acts as Restriction Factor for Human Cytomegalovirus Replication
Source: PLoS Pathog. 2012 Jan 26;8(1):e1002498. doi: 10.1371/journal.ppat.1002498 (PMC3266931; doi:10.1371/journal.ppat.1002498)
Supplement: Table S1 — Oligonucleotide primer sequences. (DOC) [file ppat.1002498.s003.doc]

**Supplemental Table S1.** Oligonucleotide primer sequences

| **Primer designation** | **Sequence (5’ to 3’)^a, b^** |
| --- | --- |
| Hs_IFI16_4 HP SI00445697 | CACGTTGAAACCAAGACTGAA |
| Hs_IFI16_6 HP SI04156005 | CAGTGGCCAAATGTCAGGTAA |
| Hs_IFI16_7 HP SI04341092 | TAGCGTTTCTGGAGATTACAA |
| Hs_IFI16_8 HP SI04373726 | CCAGCACTAGTCAGCTAACTA |
| Hs_IFNB1_1 HP SI00033621 | CAGGTTACCTCCGAAACTGAA |
| Hs_IFNB1_2 HP SI00033628 | CAGGTAGTAGGCGACACTGTT |
| Hs_IFNB1_3 HP SI00033635 | CAGAGTGGAAATCCTAAGGAA |
| Hs_IFNB1_4 HP SI00033642 | CAAGGACAGGATGAACTTTGA |
| UL54 forward | AGTCATAAGCTTTCAGACGACGGT |
| UL54 reverse | CTCGAGGGTACCGAATTCAACTCG |
| UL54-0.3 reverse | CTCGAGGGTACCCGTGGGTGGCGCGG |
| UL54-0.15 reverse | CTCGAGGGTACCCGGCGTCTCTGCATGCA |
| IR-1 mut | CCAAAGACACGTCGTTACTCTAGAATAAGGACTGAGGAAAGTGTTT |
| ATF/DR-1 mut | CTGGTGTCCGCCGACGGGCAACACGTGAATTCGGGCAACACCAAAGACACGTCGTTAC |
| MIEP forward | ACTGATGCTAGCGCATACGTTGTATCCATATC |
| MIEP reverse | AGTGATAAGCTTGCGTCTCCAGGCGAT |
| FOR_UL44 | CTCGAGAAGCTTCCGCAGCGCCAGCG |
| REV 3T600_UL44 | AGTCATAGATCTCTCTCCATCGCCAGCG |
| REV 3T160_UL44 | AGTCATAGATCTCTGGCGCTTTAAGGTCGG |
| FOR 1T600_UL44 | CTCGAGAAGCTTCAGCCGCGCCGAGC |
| IR1 wt forward | TCGTAGGAAGGCGGAGCCTGTAACGACGTG |
| IR1 wt reverse | CACGTCGTTACAGGCTCCGCCTTCCTACGA |
| IR1 mut forward | CTCGTAG**tccttattctaga**GTAACGACGT |
| IR1 mut reverse | ACGTCGTTAC**tctagaataagga**CTACGAG |
| IE1 forward | CACGACGTTCCTGCAGACTA |
| IE1 reverse | TTTTCAGCATGTGCTCCTTG |
| IE2 forward | CACTCTTGCAGCGTTAGCAG |
| IE2 reverse | CACTCTTGCAGCGTTAGCAG |
| IE2 forward | TGTTGCGGTACTGGATGGTA |
| UL44 forward | GTGGAAACTGACGCGGTTAT |
| UL44 reverse | ATCTAGATTTCGGCGTGGTG |
| UL54 forward | CGGCTACAGTATCTGCGTCA |
| UL54 reverse | AGCCACCAGGTCAGAGACAT |
| UL83 forward | GTCCTCTTCCACGTCAGAGC |
| UL83 reverse | GCAGAACCAGTGGAAAGAGC |
| β-actin forward | GTTGCTATCCAGGCTGTG |
| β-actin reverse | TGTCCACGTCACACTTCA |
| IFN−β forward | CATTAACCTGAAGGCCAAGGA |
| IFN− β  reverse | CAGCATCTGCTGGTTGAAGA |
| CCL20 forward | CCAAGAGTTTGCTCCTGGCT |
| CCL20 reverse | TGCTTGCTGCTTCTGATTCG |
| ICAM-1 forward | CAACCGGAAGGTGTATGAAC |
| ICAM-1 reverse | CAGCGTAGGGTAAGGTTC |
| IL-8 forward | ATGACTTCCAAGCTGGCCGTGGCT |
| IL-8 reverse | TCTCAGCCCTCTTCAAAAACTTCTC |

^a^Underlined letters indicate restriction enzyme sites

^b^Lowercase boldface letters indicates mutated nucleotides
